# Improving Grain Yield via Promotion of Kernel Weight in High Yielding Winter Wheat Genotypes

**DOI:** 10.3390/biology11010042

**Published:** 2021-12-29

**Authors:** Cong Zhang, Bangyou Zheng, Yong He

**Affiliations:** 1 Institute of Environment and Sustainable Development in Agriculture, Chinese Academy of Agricultural Sciences (CAAS), 12 Zhongguancun South Str., Beijing 100081, China; zhang4621792@163.com; 2 CSIRO Agriculture and Food, Queensland Biosciences Precinct, St. Lucia, Brisbane, QLD 4067, Australia; bangyou.zheng@csiro.au

**Keywords:** wheat, grain yield, kernel weight, photosynthetic rate, water-soluble carbohydrate

## Abstract

**Simple Summary:**

Improving plant net photosynthetic rates and accelerating water-soluble carbohydrate accumulation play an important role in increasing the carbon sources for wheat kernel growth and yield. The objective of this study was to quantify the relative yield contribution by analyzing the photosynthesis rate of flag leaf, water-soluble carbohydrate content of flag leaf, flag leaf sheath and stem, and other agronomic and physiological traits in 15 wheat cultivars released in Shandong Province, China between 1969 and 2006. Our results suggest that increase of flag leaf photosynthesis and WSC had a positive effect of 0.593 on the TKW, and thus benefit for developing high yielding wheat cultivars.

**Abstract:**

Improving plant net photosynthetic rates and accelerating water-soluble carbohydrate accumulation play an important role in increasing the carbon sources for yield formation of wheat (*Triticum aestivum* L.). Understanding and quantify the contribution of these traits to grain yield can provide a pathway towards increasing the yield potential of wheat. The objective of this study was to identify kernel weight gap for improving grain yield in 15 winter wheat genotypes grown in Shandong Province, China. A cluster analysis was conducted to classify the 15 wheat genotypes into high yielding (HY) and low yielding (LY) groups based on their performance in grain yield, harvest index, photosynthetic rate, kernels per square meter, and spikes per square meter from two years of field testing. While the grain yield was significantly higher in the HY group, its thousand kernel weight (TKW) was 8.8% lower than that of the LY group (*p* < 0.05). A structural equation model revealed that 83% of the total variation in grain yield for the HY group could be mainly explained by TKW, the flag leaf photosynthesis rate at the grain filling stage (Pn75), and flag leaf water-soluble carbohydrate content (WSC) at grain filling stage. Their effect values on yield were 0.579, 0.759, and 0.444, respectively. Our results suggest that increase of flag leaf photosynthesis and WSC could improve the TKW, and thus benefit for developing high yielding wheat cultivars.

## 1. Introduction

Wheat (*Triticum aestivum* L.) is the most important staple grain crop around the world. Improving yield per unit area can help achieve a steady increase in total grain output to meet the growing demand [1]. Wheat production has greatly increased since the Green Revolution because of genetic improvements, locally adapted cultivars, and enhanced management practices [2,3]. With benefit cultivar renewal, the mean genetic gains in wheat were ~1% in the United States between 1950 and 2008 [4], 1.28% in Brazil between 1984 and 2014 [5], 1.17% in Argentina between 1940 and 1999 [6], and 0.88% in Spain between 1930 and 2000 [7]. Wu et al. [8] used pooled data of all Chinese wheat zones, found that absolute yield gain and relative yield gain were 66 kg ha^−1^ yr^−1^ and 0.99% per year, respectively from 1945 to 2004. However, these improvements are considerably smaller than those needed to meet the demand for food in the mid-21st century [9,10]. Because of a decline in advancements in the field of plant genetics, economic or regulatory changes in agricultural management, and unfavorable environmental conditions [11]. Stagnation of genetic gains obtained via breeding has been reported for wheat, a trend towards the stagnation of genetic gains has been reported in several producing regions. Valvo et al. [6] indicated that Argentine cultivars released after 1999 achieved a genetic gain of only 14 kg ha^−1^ yr^−1^ (0.18% yr^−1^). In China, Sun et al. [12] observed that while wheat yields increased from 1980 to 2010 in 53% of the 1414 counties of their study, yields were found to be stagnating in 32% of the considered counties, to have never improved in 11% of the counties and to be declining in the 4% remaining counties. Similar features are also reported in Chile, for which the genetic gains obtained through breeding have plateaued since the 1990s [13]. Therefore, it is clear that the current average wheat yield requires further improvement.

Continuous selection of elite traits during wheat cultivar improvement permits the conservation and enhancement of desirable traits such as prolonged active photosynthetic duration, improved photosynthetic rates (Pn) [14] and, by extension, augmented photosynthate transport and accumulation [15] and higher grain yield. Pn has been shown to be related to yield improvement in high-yield cultivars [16,17,18,19,20,21,22,23]. In wheat cultivar renewal, Pn varies with growth stage. In old cultivars, it is relatively higher at jointing stage, whereas in modern cultivars with prolonged high Pn, it is markedly higher at later growth stages [24]. Some studies of photosynthate distribution suggest that dry matter in wheat grains mainly originates from photosynthate produced after anthesis [16,25,26,27]. The photosynthetic capacity of the flag leaves has greatly contributed to the final yield for the population [28,29]. However, as efforts have been directed toward screening germplasms for high Pn at leaf-level, and selection here has often been at the expense of other traits [23]. For example, selection for high leaf Pn has inadvertently selected for lower total leaf area because increases in leaf area may often be achieved by decreased investment per unit leaf area, light-saturated photosynthetic rate is commonly lower in species with thinner leaves [21]. Therefore, traditional breeding and selection approaches for higher yield have only resulted in small improvements in the photosynthetic efficiency, photosynthetic potential merits further investigation [30].

Water soluble carbohydrates (WSC) stored in stems and leaf sheaths contribute to grain growth as the major carbon resource for grain yield [31,32,33,34,35,36,37,38,39]. The contribution of WSC to yield varies greatly with environments, cultivars, and organs. Some studies have shown that WSC in wheat are mobilized from the stem and leaf sheath during the later phase of grain filling and can potentially contribute to about 20% of grain yield under normal conditions [40,41]. WSC could potentially contribute to ~50% of grain yield [32,42] while under terminal drought stress conditions, because the carbon supply from photosynthesis is reduced during drought stress due to both stomatal closure in the leaves [43] and coordinated down-regulation of genes involved in the Calvin cycle [44]. The study of Shearman et al. [41] showed significant genetic changes across time and correlations with grain yield were found for WSC content of stems and leaf sheaths at anthesis (4.6 g m^−2^ yr^−1^). Dreccer et al. [45] demonstrated that the grain filling rate, grain weight, and yield in high WSC content cultivars increased by 41%, 34%, and 10% relative to lower WSC content cultivars, respectively. Rebetzke et al. [36] reported a dramatic difference between wheat leaves and stems in terms of the WSC content. Yang et al. [46] found that the WSC content of the whole stem during mid grain filling plays a key function in the subsequent release of carbohydrates from stem to grain, had extremely significant positive correlations with thousand-kernel weight (TKW) and yield. High WSC accumulation and effective WSC distribution at various growth stages and in different organs are required for elevated grain yield. For this, the ability to store and remobilize large amounts of stems and leaf, leaf sheaths WSC constitutes a seemingly desirable trait to incorporate in germplasm.

Wheat yield-related traits are all complex agronomic and physiological traits [47], integration of traits such as the source–sink balance must be considered to achieve successful grain yield gain [48,49]. Sink size in developing yield organs is determined by the number of spikes per unit area, grains per spike, sink size per grain, and thousand-grain weight. Source size is related to photosynthate production, leaf area, photosynthesis rate, and duration [50,51]. The source restores and supplies reserves to the sink. Source–sink affect yield formation from the first visible spikelet ridges to the end of grain filling. Wheat productivity is generally considered to be sink-limited under favorable conditions, with grain development regulated by the assimilating capacity but hardly limited by the source, which assimilates to developing grains because the source generally has the capacity [52,53]. Slafer and Savin [54] pointed out that during the post-anthesis period, grain yield of wheat is either sink-limited or co-limited by both source and sink but never source-limited. Miralles and Slafer [55] found that wheat yield is limited mainly by sink-strength during grain filling, although, as the number of grains is increased, a sort of source-sink colimitation during grain filling. Rivera-Amado et al. [56] noticed that grain growth among 26 elite spring wheat cultivars has been influenced form sink limitation to probable source and sink colimitation, this may have been due to an increase in grain sink strength with years of cultivar release with no commensurate increase in post-anthesis source capacity. Recently, Burnett et al. [57] has presented that whilst increased source can be shown to enhance yield, the response is often not matched by an equivalent increase in plant growth and yield (sink). Thus, the finely tuned balance between sources and sinks is crucial for resource partitioning and regulates growth and yield.

Agronomic and physiological performance levels are the direct readouts of the genetic traits of a cultivar. It is crucial to identify the individual factors and their interactions influencing yield in wheat breeding. To this end, (1) experiments must be conducted under field conditions, (2) measurements must be made in comparable field plots [58], (3) cultivars released at various times must be compared simultaneously [59], and (4) the statistical techniques used must be able to characterize the synergistic contributions of multiple agronomic and physiological traits to grain yield. Shandong Province ranks first in agricultural production [60], which plays an important role in securing food supply in China [61]. The total sown area in Shandong Province amounted to 11.10 million hectares in 2017 and accounted for almost 7% of national total. The production of wheat was 24.95 million tons in 2017, which was 19% of national totals [62]. Therefore, research on genetic wheat yield improvement in Shandong Province is vital to both regional and national wheat breeding and production. The potential for yield improvement in HY wheat cultivars may mainly from increased TKW, that is, by increasing Pn to improve the WSC required for grain filling. So far, however, studies to understand how to improve grain yield via promotion of kernel weight in high yielding winter wheat genotypes in this province are still rare in literature. The objectives of this study were to (1) quantify the contribution of photosynthetic rate and water-soluble carbohydrate to grain yield and yield components of 15 genotypes released from 1969–2006, (2) identify the relationship between sink and source related traits in HY and LY wheat cultivars, and (3) address the factors in current wheat breeding strategies that should be ameliorated to increase TKW and grain yield in HY wheat cultivars for Shandong Province located at the Northern China Winter-Wheat Region.

## 2. Materials and Methods

### 2.1. Field Conditions and Experimental Design

Experiments were conducted during three successive crop seasons (2006–2007, 2007–2008, and 2008–2009) at the Tai’an Academy of Agricultural Science (36°11′5″ N, 117°10′58″ E, and 153 m a·s·l.) in central Shandong Province, China. This semi-humid region has a warm temperate continental monsoon climate. Monthly average precipitation and temperatures for the 2007–2009 wheat growing seasons and the wheat growth data for 2001–2009 were obtained from the Agricultural Meteorological Observatory (Figure S1). The soil type was a clay loam with, on average, 18.2 g·kg^−1^ organic matter, 1.4 g·kg^−1^ total N, 156.7 mg·kg^−1^ available N, 24.6 mg·kg^−1^ available P, and 120.6 mg·kg^−1^ available K in the 0–20 cm soil depth.

Plant materials and experimental design details were previously described [63]. Hence, only the information that is directly pertinent to the present study is reported here. There were 15 winter wheat genotypes, including 13 milestone cultivars and two advanced lines (Table S1). They represented significant progress in wheat breeding and were selected to reflect the most historically important cultivars in Shandong, Henan, Hebei, and Shanxi between 1969 and 2006 [63]. These genotypes were highly adaptable, resilient, and resistant to natural disasters. A randomized complete block design with three replications was used and the genotypes were planted in replicated plots on 11 October 2006, 15 October 2007, and 9 October 2008 at a seeding rate of 250 seeds·m^−2^. This irrigated high-yield region is sown annually with a wheat–maize rotation. Six agronomic traits (KnSq, TKW, KnSpk, SpkSq, KwSpk, and HI) and 12 physiological traits (Chl65, Pn55, Pn65, Pn75, CTD65, CTD75, LWSC65, ShWSC65, StWSC65, LWSC75, ShWSC75, and StWSC75) were evaluated (Table S2). Physiological traits were mainly measured at heading, anthesis and grain filling stages; heading and anthesis are important turning points of wheat growth, and the grain filling stage is the key stage of kernel growth. In the present study, only the experimental data for the 2007–2009 growing seasons were analyzed.

### 2.2. Statistical Analysis

A cluster analysis was performed using the “cluster” and “vegan” packages in R software Version 3.6.2 (2019-12-12, R Foundation for Statistical Computing, Vienna, Austria. http://www.R-project.org/, accessed on 20 December 2021), within this analysis the 15 genotypes were grouped as LY or HY group by their similarities of data (yield, physiological and agronomic traits, see Table S2 for details). A non-parametric multi-response permutation procedure analysis tested the differences between the LY and HY groups. Tukey’s test was used to analyze differences between three stages of Pn, different lowercase letters in the figure indicate significant differences. Student’s *t*-test and Wilcoxon rank sum test were used to compare yield and yield components among the LY and HY groups. Student’s *t*-test was selected if the data fulfill requirements of normality and homogeneity of variance, otherwise the Wilcox-test was used. Pearson’s coefficients of correlation among yield components, HI, and yield were calculated using the “GGally” package in R [64]. Significance was accepted at *p* < 0.05. A structural equation model (SEM) analysis was performed using the “lavaan” package in R to quantify the relationships among the dependent variables and independent variables [65,66]. SEM is generalized of path analysis proposed by Wright [67], a biostatistician, in the early 1920s. And SEM is used to quantitatively analyze the causal structure among a number of variables where each may function as a dependent variable in some equations and an independent variable in others [68]. All SEM path coefficients were subjected to standardized partial regression coefficients measuring the direct influence of each independent variable on the dependent variable. This approach separated the path coefficients into direct and indirect effect components [69,70].

## 3. Results

### 3.1. HY Group Has Larger Sink than LY Group

**Figure 1:**
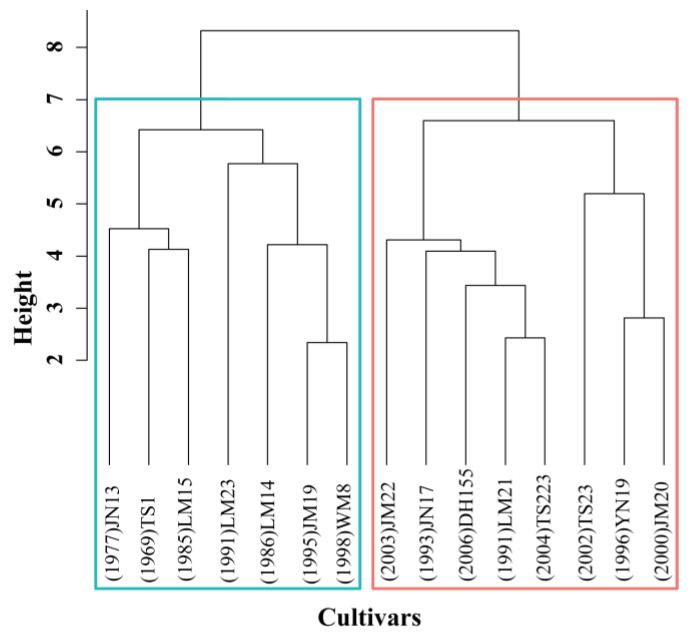
Similarity among 15 wheat cultivars estimated by hierarchical cluster analysis. Abscissa: wheat cultivar clustering categories. Each vertical line represents one category. Ordinate: relative distance of each category. Cluster 1 is outlined in blue and comprises JN13 (Jinan13), TS1 (Taishan1), LM15 (Lumai15), LM23 (Lumai23), LM24 (Lumai14), JM19 (Jimai19), and WM8 (Weimai8). Cluster 2 is outlined in red and comprises JM22 (Jimai22), JN17 (Jinan17), DH155 (Zhongmai155), LM21 (Lumai21), TS223 (Taishan223), TS23 (Taishan23), YN19 (Yannong19), and JM20 (Jimai20).

**Figure 2:**
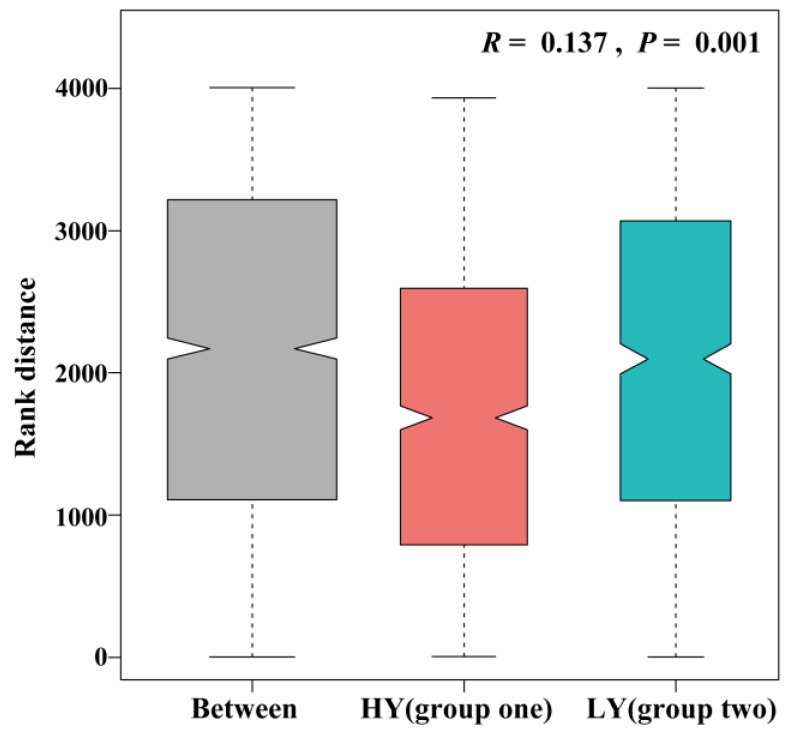
Similarity analysis of the LY and HY groups wheat cultivars. Box plots showing rank distributions between (Between) and within (LY and HY) groups. Y-axis represents rank distance. Grooves on both sides of boxes represent confidence intervals of median. Upper and lower box edges represent 25th and 75th percentiles of all data, respectively. Lengths of lines at the bottom and top represent 5th and 95th percentiles, respectively. *R* (ANOSIM statistic R) indicates degree of difference between and within groups, where the range is (−1, 1). *R* > 0 indicates that the differences between groups are significant and greater than those within groups. *p* < 0.05 indicates statistical significance. LY and HY groups are Cluster 1 and Cluster 2 in Figure 1, respectively.

Grain yield, yield components, and physiological traits were used as input variables in a cluster analysis to classify the 15 genotypes as two groups. According to their performance of gain yield, two groups were roughly recorded as low yielding (LY) group and high yielding (HY) group. There were seven genotypes in LY group, including LM23, that were mainly released before the 21st century. There were eight genotypes in HY group, including JM20, that were mainly released in the 21st century (Figure 1). A similarity analysis (Figure 2) revealed that compositional dissimilarities between the groups were greater than those within the groups (*R* = 0.137, *p* = 0.001), indicating that the LY and HY groups were significantly different.

**Figure 3:**
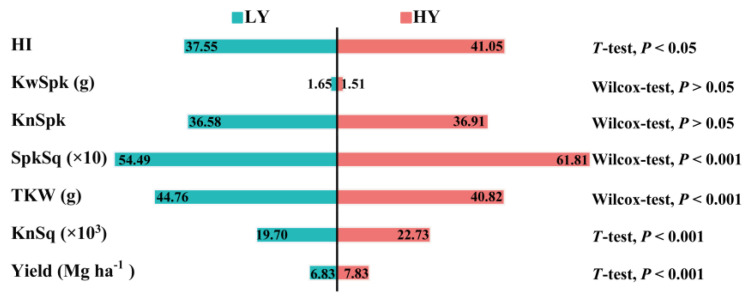
Comparisons of winter wheat grain yield (Yield), kernels per square meter (KnSq), thousand-kernel weight (TKW), spikes per square meter (SpkSq), kernels per spike (KnSpk), kernel weight per spike (KwSpk), and harvest index (HI). Fitted data were 2-year means for each genotype released between 1969 and 2006. *T*-test: Student’s *t*-test; Wilcox-test: Wilcoxon rank-sum test; *p* < 0.001: extremely significant difference; *p* < 0.05: significant difference; *p* > 0.05: no significant difference. Abbreviations are explained in Table S2. LY and HY groups are shown in Figure 1.

*T*-tests and non-parametric tests were conducted to compare yield, harvest index (HI), and yield components between the LY and HY groups (Figure 3). Grain yield, kernels per square meter (KnSq), and spikes per square meter (SpkSq) were extremely significantly (*p* < 0.001) higher for the HY than for the LY group. The HI of HY group was also significant (*p* < 0.05) higher. In contrast, thousand-kernel weight (TKW) of HY group was extremely significantly lower than the LY group (*p* < 0.001). Neither kernel weight per spike (KwSpk) nor kernels per spike (KnSpk) significantly differed between LY and HY groups. Thus, the HY group has a large sink compared to the LY group.

### 3.2. TKW, Pn and WSC Have Substantial Effects on HY Group Yield

**Figure 4:**
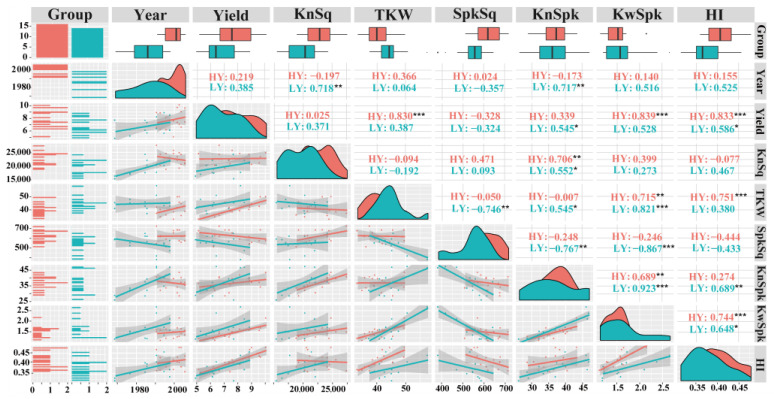
Correlation matrices for grain yield (Yield), kernels per square meter (KnSq), thousand-kernel weight (TKW), spikes per square meter (SpkSq), kernels per spike (KnSpk), kernel weight per spike (KwSpk), and harvest index (HI) for seven LY group (blue) and eight HY group (red). HY and LY groups are shown in Figure 1. *** *p* < 0.001; ** *p* < 0.01; * *p* < 0.05.

A Pearson correlation analysis of yield, HI, and yield components was shown in Figure 4. Grain yield was positively correlated with HI and KnSpk for LY group, while positively correlated with TKW, KwSpk, and HI for HY group. Due to a high degree of collinearity among KwSpk, KnSpk, and TKW, only KnSq, TKW, and HI were selected for the subsequent analyses.

**Figure 5:**
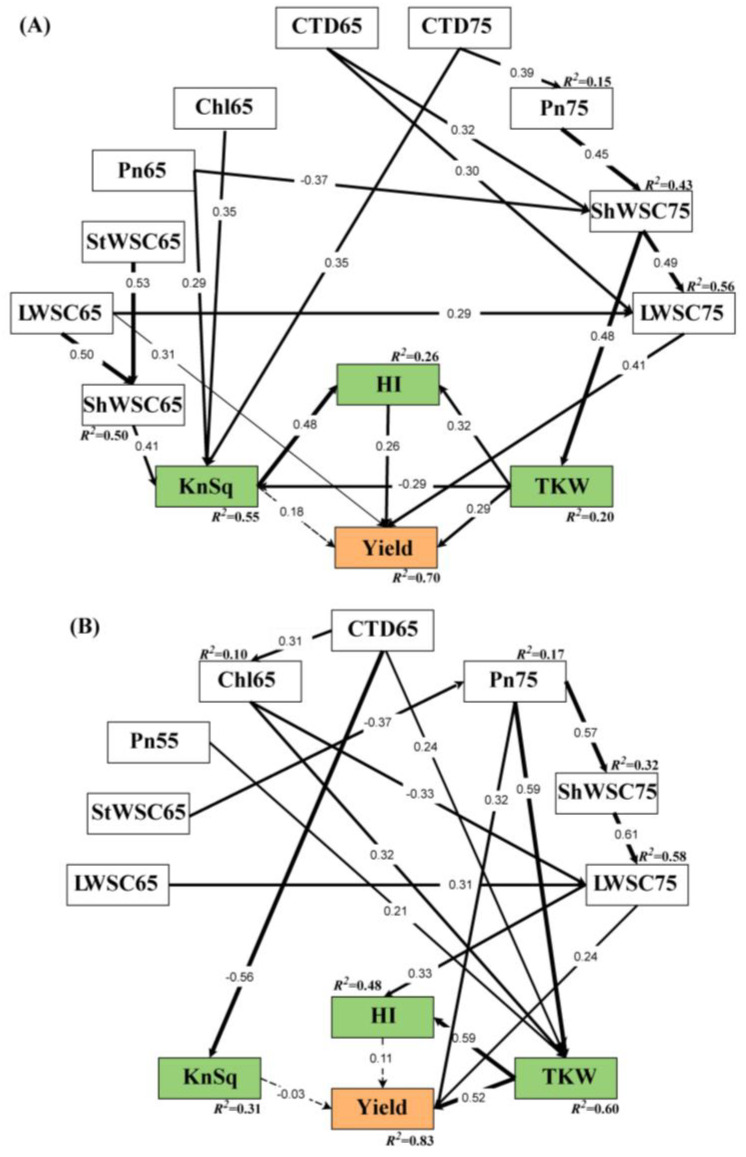
Structural equation modeling (SEM) showing effects of yield, kernels per square meter (KnSq), thousand-kernel weight (TKW), and harvest index (HI). Contributions of photosynthetic rate (Pn), chlorophyll content (Chl), canopy temperature depression (CTD), water-soluble carbohydrate in flag leaf (LWSC), water-soluble carbohydrate in stem (StWSC), and water-soluble carbohydrate in flag leaf sheath (ShWSC) at heading (DC55), anthesis (DC65), and grain filling stage (DC75) to grain yield. Black lines and arrows indicate significant pathways (*p* < 0.05), with line thickness representing significance. Progressively greater significance levels are represented by increasing thicknesses of the solid lines. No line or dotted lines indicate non-significant paths (*p* > 0.05). Coefficients indicate the strength of the association between variables, while arrows indicate directionality. R^2^ Values indicate the proportion of total variance for a variable explained by the model. For the seven LY group (**A**), x^2^/df = 1.054; *p* = 0.367; GFI = 0.819; CFI = 0.984; RMSEA < 0.05. For the eight HY group (**B**), x^2^/df = 1.199; *p* = 0.176; GFI = 0.829; CFI = 0.966; RMSEA < 0.05. Abbreviations are explained in Table S2. HY and LY groups are shown in Figure 1.

SEM analysis was performed (Figure 5) to quantify the relationships among photosynthetic rate (Pn) of flag leaves, chlorophyll content (Chl), canopy temperature depression (CTD), water-soluble carbohydrate (WSC), KnSq, TKW, HI, and grain yield (Figure 5). We show the standardized direct and indirect effects of LY group (Table 1) and HY group (Table 2); the standardized direct effects plus indirect effects are the total effects. In the LY group, the following factors had a strong total effect on the grain yield: KnSq, TKW, HI, LWSC65 (water-soluble carbohydrate in flag leaf at anthesis; Zadoks = 65), LWSC75 (water-soluble carbohydrate in flag leaf at grain filling stage; Zadoks = 75), and ShWSC75 (water-soluble carbohydrate in flag leaf sheath at grain filling stage; Zadoks = 75). KnSq had a larger total effect on grain yield than TKW or HI. LWSC65 had a direct effect on wheat grain yield and its standardized path coefficient was 0.31 (*p* < 0.001). It indirectly contributed to yield via KnSq and HI. ShWSC75 affected yield mainly through LWSC75 and TKW. LWSC75 had a strong significant direct effect on wheat grain yield. KnSq had no significant effect on yield but its standardized path coefficient for HI was 0.48 (*p* < 0.001) and it made a highly significant contribution to HI. TKW significantly and directly affected wheat grain yield and indirectly influenced it via HI. The SEM demonstrated that KnSq was influenced mainly by WSC, Pn, Chl, and CTD. Their cumulative contribution rate was 0.55 (*p* < 0.001). TKW was affected primarily through WSC, and its cumulative contribution rate was comparatively lower.

For the HY group, the total effects of LWSC75, StWSC65 (peduncle WSC at anthesis), and especially Pn75 (Pn at grain filling stage) and TKW on yield were relatively higher than other traits. Their direct standardized path coefficients for yield were 0.32 (*p* < 0.001) and 0.52 (*p* < 0.001), respectively. Pn75 also indirectly influenced yield via LWSC75 and TKW. LWSC75 was directly affected through ShWSC75. This interaction highly and significantly affected the grain yield and indirectly influenced it through HI. For the HY group, TKW was significantly affected by Pn75, Chl, and CTD, and all of these variables collectively contributed to the grain yield.

The main variables influencing grain yield differed between the HY and LY groups. LWSC65, LWSC75, ShWSC75, HI, KnSq, and TKW were the main variables influencing the LY group grain yield. In contrast, Pn75, TKW, and LWSC75 were the main variables influencing the HY group yield. During cultivar improvement, yield gain depended primarily on the increase in TKW, which was, in turn, affected by Pn75 and LWSC75. LWSC75, ShWSC75, TKW, and Pn75 were relatively stable and were the main factors influencing grain yields.

### 3.3. HY Group Had Elevated Pn and Prolonged Active Photographic Duration

**Figure 6:**
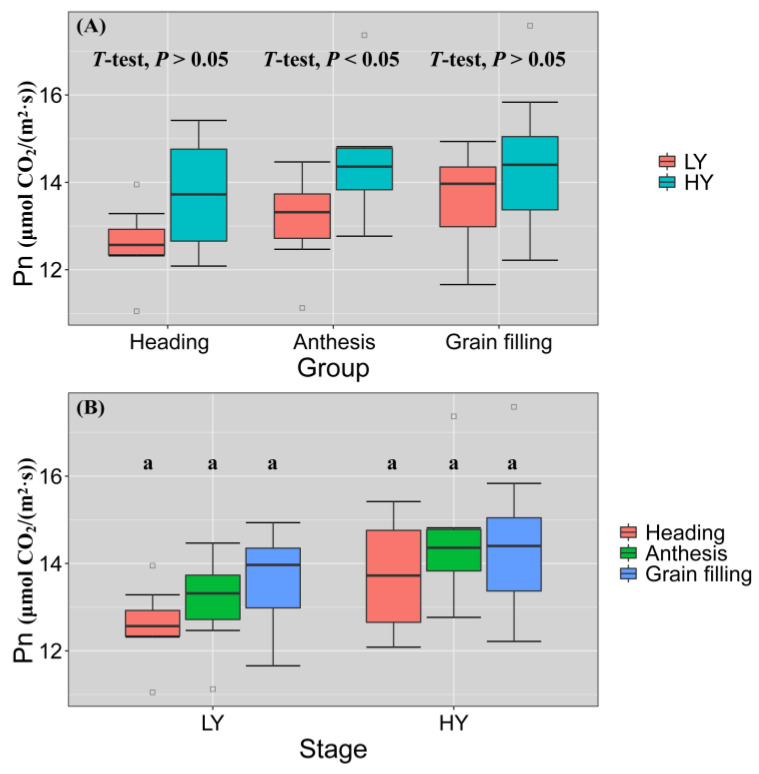
Flag leaf photosynthetic rates (Pn) for seven LY group cultivars and eight HY group cultivars (**B**) at heading (DC55), anthesis (DC65), and grain filling (DC75) stages (**A**). Solid lines within boxes indicate medians. Upper and lower box edges represent 25th and 75th percentiles of all data. Lengths of lines at bottom and top represent 5th and 95th percentiles, respectively. HY and LY wheat cultivars are shown in Figure 1. *T*-test: Student’s *t*-test; Wilcox-test: Wilcoxon rank-sum test; *p* < 0.001: extremely significant difference; *p* < 0.05: significant difference; *p* > 0.05: no significant difference. Similar letters (a) indicate no significant differences among three stage means for Pn (α = 0.05, Tukey’s test).

SEM analysis demonstrated that Pn of flag leaves plays an important role in yield improvement. We used box plots to analyze the differences in Pn of flag leaves between the LY and HY groups at various stages (Figure 6). In the LY group, the mean values of Pn at the heading stage, flowering stage, and filling stage were 12.586, 13.116, and 13.604, respectively, while in the HY group, they were 13.700, 14.473, and 14.429. There was no significant difference in Pn between three stages of LY and HY group. At three stages, especially at anthesis, the Pn was higher for the HY group than for the LY group and the differences were significant (*p* < 0.05). However, there were no significant differences in Pn between LY and HY groups at heading or grain filling stage. The Pn of the LY group increased from heading to grain filling stage and the median range was 12.567–13.964. In contrast, the Pn of the HY group peaked at anthesis (median = 14.159) but remained high at filling stage (median = 14.217). Hence, the HY group had prolonged active photographic duration than the LY group.

### 3.4. HY Group Had Lower WSC

**Figure 7:**
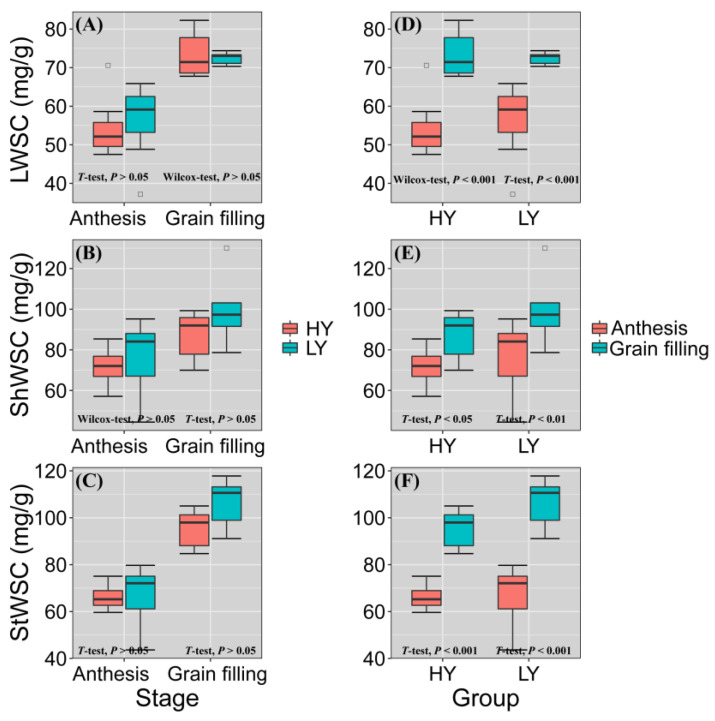
Water-soluble carbohydrate content in flag leaf (LWSC) (**A**,**D**), flag leaf sheath (ShWSC) (**B**,**E**), and stem (StWSC) (**C**,**F**) at heading (DC55), anthesis (DC65), and grain filling (DC75) stages for seven LY cultivars and eight HY cultivars. Solid lines within boxes indicate medians. Upper and lower box edges represent 25th and 75th percentiles of all data. Lengths of lines at bottom and top represent 5th and 95th percentiles, respectively. HY and LY groups are shown in Figure 1. *T*-test: Student’s *t*-test; Wilcox-test: Wilcoxon rank-sum test; *p* < 0.001: extremely significant difference; *p* < 0.05: significant difference; *p* > 0.05: no significant difference.

The WSC content in flag leaf and leaf sheath strongly affect yield. To clarify the effects of WSC on yield, we compared WSC content among various organs of HY and LY groups at anthesis and the grain filling stage (Figure 7). Overall, the WSC content in the flag leaf, leaf sheath, and stem of the HY group was lower than in those of the LY group. Moreover, WSC increased significantly from flowering to grain filling stage, especially in flag leaf (*p* < 0.001) and stem (*p* < 0.001). At anthesis, the order of WSC content was flag leaf < stem < flag leaf sheath. At grain filling stage, the order of WSC content was flag leaf < flag leaf sheath < stem. The order of the rate of WSC increase was stem > flag leaf > flag leaf sheath at both anthesis and grain filling stage. WSC of stem increased most from flowering to filling stage.

## 4. Discussion

Higher yield levels can be achieved by accelerating and sustaining post-anthesis photosynthesis and WSC [22,71,72,73]. So far, numerous studies have been conducted to quantify to contribution of these traits for yield improvement. However, how to coordinate and optimize these two characteristics to balance the sink and source is still unclear [74,75]. In this study, we found that the HY group had higher Pn. Moreover, the period for high value of Pn lasted longer in HY group than LY group (Figure 6). These are consistent with research of Wang et al. [76]. They found that the flag leaf photosynthetic rate showed an increasing trend with the advance of yield, and new cultivars had higher Pn, and the high value of Pn lasted for a long time. In addition, our present study demonstrated that the total effect of Pn75 on grain yield was substantially greater in the HY group (0.759) than in the LY group (0.153). Hence, increased Pn75 made a great contribution to yield gain. This finding was consistent with those reported in previous studies [77]. Pn is a vital source trait, increasing it can enhance source assimilate supply and ensure adequate assimilate output. However, Pn falls far short of its biological limit; there is a significant underutilized photosynthetic capacity among existing wheat varieties, emerging as the key remaining route to increase the genetic yield potential [23,78]. Gaju et al. [79] reported leaf Pn of modern wheat cultivars can be improved by crossing with wild relatives. Improvement of photosynthetic performance and its benefits to yield must be considered in terms of the canopy rather than a single leaf [80]. Therefore, the leaf areas, leaf area indices, and surface areas of non-leaf organs may be augmented by breeding, cultivation, and fertilizer and irrigation control [81,82,83,84].

Water soluble carbohydrates (WSC) stored in the stems and leaf sheaths contribute to grain growth as the major carbon resource [85,86]. Consequently, WSC are a major contributor to wheat grain yield and grain size in all environments [41]. We found that WSC content in the flag leaf and flag leaf sheath had a considerable contribution to yield in both LY and HY groups, and HY group had lower WSC by comparison. which showed that the carbon source of HY group grain filling was relatively insufficient. In addition, WSC content and its effect on yield varied with plant organ and growth period [87,88]. In the present study, the rate of change in WSC content from anthesis to grain filling stage was in the order stem > flag leaf > flag leaf sheath. At grain filling stage, the WSC content was the highest in the stem and the lowest in the flag leaf. These results were consistent with those of previous studies. Takahashi et al. [89] showed that leaves and leaf sheaths are short-term WSC storage organs and that WSC were stored during the day and transported at night, showed the peak of WSC contents at about anthesis. The stem internodes were long-term WSC storage organs, accumulated WSC during early grain filling phase, from a week after anthesis until the milk-ripe stage, then remobilized them during late and final grain filling phases, about 2–3 weeks post-anthesis. In the present study, 14 days after anthesis was considered the grain filling stage. At this time, the plant is actively photosynthesizing [90], the current photosynthate provides sufficient carbohydrate for grain filling so that surplus WSC are accumulated in stem. In fact, some WSC are stored during this period. For these reasons, StWSC75 was relatively high. As the distance from stems to grains is short, it is relatively easy to transport carbohydrates from the former to the latter during late filling. Future wheat breeding initiatives should focus on enhancing WSC accumulation and transport from both the flag leaves and the stems to the grains.

Increasing the sink during cultivar improvement has greatly improved grain yield. Comparisons of the yield, HI, and yield components of HY and LY cultivars revealed that there was no significant difference in KnSpk between LY and HY groups, but the HY group had extremely significantly higher SpkSq than the LY group. Namely, the storage capacity of HY group per unit area increased, so the yield increased. This also means that more WSC are needed to maintain plant growth, while the supply of WSC for grain filling may be insufficient. Meanwhile, from the lower TKW and WSC of HY group, it can be seen that increase in grain sink with no commensurate increase in post-anthesis source capacity [56]. So, the source should be increased in the future [56,74].

In response to cultivar improvement, TKW as the main variable influencing yield, which fit with others studies [8,9,18,91,92,93]. The present study showed that KnSq was significantly correlated with yield in LY group, whereas TKW was the most relevant yield-influencing trait in HY group. Wang et al. [94] pointed out that during wheat cultivar improvement in Shandong, the ranking of factor contribution to grain yield changed from KnSpk > SpkSq > TKW in the 1950s to TKW > KnSpk > SpkSq for modern cultivars. Calderini et al. [95] analyzed local cultivars released between 1920 and 1990 and observed that they had different trends in grain weight. Yield gain was a consequence of incremental increases in grain number for cultivars released before the 1980s. In contrast, between the late 1980s and the 1990s, incremental increases in grain weight determined yield gain. Similar findings were reported by Sadras and Lawson [96]. Therefore, increasing TKW while ensuring grain number is a feasible choice for increasing yield. This is supported by wheat breeding and genetic research has shown that it is possible to increase grain size without a negative effect on grain number [97] by genetic manipulation of grain expansion [98].

These results test our hypothesis, showing improvements in grain yield mainly via promotion of kernel weight through increasing Pn and WSC in high yielding winter wheat genotypes, on the basis of sufficient population.

## 5. Conclusions

Genetic gains in wheat yield are driven by integrating the photosynthesis rate with yield-related agronomic traits to develop genotypes with higher yield potential. Compared with LY group, the HY group had larger sinks but smaller, inadequate sources, indicating that the source–sink relationship was not coordinated. We highlight the importance of coordinating and optimizing these characteristics to balance the sink and source for achieving a high yield. As a result, all photosynthetic canopy organs must be collectively considered, and the photosynthesis rate of the flag leaves and non-leaf organs should be evaluated [48,51,99]. In this manner, the photosynthetic capacity of the entire wheat plant and the whole plant population can be fully exploited to improve the photosynthesis rate. Increasing the WSC reserves of flag leaf, flag leaf sheath, and stem obtain a greater grain filling source, relieve carbon source limitations during kernel growth, and lead to improvements in the TKW, biological, and agronomic yield of wheat. These results provide a pathway to overcome this grain gain bottleneck in order to improve yield across crops.

New cultivars with elite characters have been constantly released. Therefore, the genotypes used in this study may not represent the latest high yielding cultivars. Nevertheless, the 15 wheat accessions examined herein were elite cultivars that have been planted in the local area for a long period of time. In addition, the data about WSC content at a late filling and mature stage in a future study would allow for measurement.

## Figures and Tables

**Table 1:** Standardized direct and indirect effects of independent variables on dependent variables of LY group in structural equation modeling. Independent variables including canopy temperature depression at heading (CTD65) and grain filling stage (CTD75), chlorophyll content at heading (Chl65), photosynthetic rate at anthesis (Pn65) and grain filling stage (Pn75), water-soluble carbohydrate in stem at anthesis (StWSC65), water-soluble carbohydrate in flag leaf at anthesis (LWSC65) and grain filling stage (LWSC75), water-soluble carbohydrate in flag leaf sheath at anthesis (ShWSC65) and grain filling stage (ShWSC75), kernels per square meter (KnSq), thousand-kernel weight (TKW), and harvest index (HI). Dependent variables including Pn75, ShWSC65, LWSC75, ShWSC75, KnSq, TKW, HI and yield.

		Pn75	ShWSC65	ShWSC75	LWSC75	TKW	KnSq	HI	Yield
Direct effects	CTD65	0.000	0.000	0.301 **	0.315 **	0.000	0.000	0.000	0.000
	CTD75	0.387 **	0.000	0.000	0.000	0.000	0.348 **	0.000	0.000
	Chl65	0.000	0.000	0.000	0.000	0.000	0.348 **	0.000	0.000
	Pn65	0.000	0.000	−0.374 ***	0.000	0.000	0.292 **	0.000	0.000
	Pn75	0.000	0.000	0.448 ***	0.000	0.000	0.000	0.000	0.000
	LWSC65	0.000	0.503 ***	0.000	0.294 **	0.000	0.000	0.000	0.315 ***
	ShWSC65	0.000	0.000	0.000	0.000	0.000	0.408 ***	0.000	0.000
	StWSC65	0.000	0.533 ***	0.000	0.000	0.000	0.000	0.000	0.000
	LWSC75	0.000	0.000	0.000	0.000	0.000	0.000	0.000	0.407 ***
	ShWSC75	0.000	0.000	0.000	0.494 ***	0.448 ***	0.000	0.000	0.000
	TKW	0.000	0.000	0.000	0.000	0.000	−0.288 **	0.319 **	0.293 **
	KnSq	0.000	0.000	0.000	0.000	0.000	0.000	0.480 ***	0.175
	HI	0.000	0.000	0.000	0.000	0.000	0.000	0.000	0.256 **
Indirect effects	CTD65	0.000	0.000	0.000	0.154	0.135	−0.037	0.024	0.235 **
	CTD75	0.000	0.000	0.173	0.089	0.078	−0.021	0.180	0.166
	Chl65	0.000	0.000	0.000	0.000	0.000	0.000	0.166	0.107
	Pn65	0.000	0.000	0.000	−0.192	−0.167	0.046	0.109	−0.038
	Pn75	0.000	0.000	0.000	0.230 **	0.200 *	−0.055	0.036	0.153
	LWSC65	0.000	0.000	0.000	0.000	0.000	0.196 *	0.098	0.186
	ShWSC65	0.000	0.000	0.000	0.000	0.000	0.000	0.202 **	0.131
	StWSC65	0.000	0.000	0.000	0.000	0.000	0.208 **	0.104	0.067
	LWSC75	0.000	0.000	0.000	0.000	0.000	0.000	0.000	0.000
	ShWSC75	0.000	0.000	0.000	0.000	0.000	−0.123	0.080	0.341 ***
	TKW	0.000	0.000	0.000	0.000	0.000	0.000	−0.137	−0.004
	KnSq	0.000	0.000	0.000	0.000	0.000	0.000	0.000	0.133
	HI	0.000	0.000	0.000	0.000	0.000	0.000	0.000	0.000

Abbreviations are explained in Table S2. LY group cultivars are shown in Figure 1. All data in this table are from SEM. Asterisks indicate significant effects: *, *p* < 0.05; **, *p* < 0.01; ***, *p* < 0.001; ‘0.000’ in this table indicates the path between variables deleted during SEM model modification due to the path coefficient is not significant (*p* > 0.05).

**Table 2:** Standardized direct and indirect effects of independent variables on dependent variables of HY group in structural equation modeling. Independent variables including canopy temperature depression at heading (CTD65), chlorophyll content at heading (Chl65), photosynthetic rate at heading (Pn55), and grain filling stage (Pn75), water-soluble carbohydrate in stem at anthesis (StWSC65), water-soluble carbohydrate in flag leaf at anthesis (LWSC65) and grain filling stage (LWSC75), water-soluble carbohydrate in flag leaf sheath at grain filling stage (ShWSC75), kernels per square meter (KnSq), thousand-kernel weight (TKW), and harvest index (HI). Dependent variables including Chl65, Pn75, LWSC75, ShWSC75, KnSq, TKW, HI, and yield.

		Chl65	Pn75	LWSC75	ShWSC75	KnSq	TKW	HI	Yield
Direct effects	CTD65	0.314 *	0.000	0.000	0.000	−0.559 ***	0.237 *	0.000	0.000
	Chl65	0.000	0.000	−0.327 ***	0.000	0.000	0.315 **	0.000	0.000
	Pn55	0.000	0.000	0.000	0.000	0.000	0.205 *	0.000	0.000
	Pn75	0.000	0.000	0.000	0.569 ***	0.000	0.593 ***	0.000	0.319 ***
	StWSC65	0.000	−0.365 **	0.000	0.000	0.000	0.000	0.000	0.000
	LWSC65	0.000	0.000	0.314 ***	0.000	0.000	0.000	0.000	0.000
	LWSC75	0.000	0.000	0.000	0.000	0.000	0.000	0.326**	0.238 **
	ShWSC75	0.000	0.000	0.615 ***	0.000	0.000	0.000	0.000	0.000
	KnSq	0.000	0.000	0.000	0.000	0.000	0.000	0.000	−0.029
	TKW	0.000	0.000	0.000	0.000	0.000	0.000	0.587 ***	0.514 ***
	HI	0.000	0.000	0.000	0.000	0.000	0.000	0.000	0.110
Indirect effects	CTD65	0.000	0.000	−0.102	0.000	0.000	0.100	0.165	0.184
	Chl65	0.000	0.000	0.000	0.000	0.000	0.000	0.079	0.094
	Pn55	0.000	0.000	0.000	0.000	0.000	0.000	0.121	0.120
	Pn75	0.000	0.000	0.349 ***	0.000	0.000	0.000	0.462 ***	0.440 ***
	StWSC65	0.000	0.000	−0.127	−0.208 **	0.000	−0.217 **	−0.169	−0.278 **
	LWSC65	0.000	0.000	0.000	0.000	0.000	0.000	0.103	0.087
	LWSC75	0.000	0.000	0.000	0.000	0.000	0.000	0.000	0.036
	ShWSC75	0.000	0.000	0.000	0.000	0.000	0.000	0.201**	0.170
	KnSq	0.000	0.000	0.000	0.000	0.000	0.000	0.000	0.000
	TKW	0.000	0.000	0.000	0.000	0.000	0.000	0.000	0.065
	HI	0.000	0.000	0.000	0.000	0.000	0.000	0.000	0.000

Abbreviations are explained in Table S2. HY group cultivars are shown in Figure 1. All data in this table are from SEM. Asterisks indicate significant effects: *, *p* < 0.05; **, *p* < 0.01; ***, *p* < 0.001; ‘0.000’ in this table indicates the path between variables deleted during SEM model modification due to the path coefficient is not significant (*p* > 0.05).

## Data Availability

All data is contained within the article or Supplementary Material.

## Supplementary Materials

The following supporting information can be downloaded at: https://www.mdpi.com/article/10.3390/biology11010042/s1, Figure S1: Monthly average temperature and monthly accumulated precipitation for the 2007–2008 and 2008–2009 growing seasons in Tai’an, Shandong, China. Bars in blue and red represent monthly cumulative precipitation during the 2007–2008 and 2008–2009 wheat growing seasons, respec-tively. Black and white circles represent monthly average temperatures during the 2007–2008 and 2008–2009 wheat growing seasons, respectively. Blue circles represent crop phenology stages on the Zadoks scale [100]. The data were derived from the China Meteorological Data Service Center website (http://data.cma.cn, accessed on 20 December 2021); Table S1: Year released, pedigree, growing period and cumulative planting area of 15 winter wheat geno-types grown in the Tai’an, Shandong province, China, between 2007 and 2009; Table S2: Agronomic and physiological traits of cultivars grown under field conditions during the 2007–2008 and 2008–2009 wheat growing seasons. The Zadoks growth scale, a numerical code representing various cereal crop growth and development stages, is provided by Zadoks et al. [100].

## Abbreviations

Chl, chlorophyll content; CTD, canopy temperature depression; HI, harvest index; KnSpk, kernels per spike; KnSq, kernels per square meter; KwSpk, kernel weight per spike; LWSC65, flag leaf WSC at the anthesis stage (Zadoks = 65); LWSC75, flag leaf WSC at the grain filling stage (Zadoks = 75); Pn, photosynthetic rate; SEM, structural equation model; ShWSC65, flag leaf sheath WSC at the anthesis stage (Zadoks = 65); ShWSC75, flag leaf sheath WSC at the grain filling stage (Zadoks = 75); SpkSq, spikes per square meter; StWSC65, stem WSC at the anthesis stage (Zadoks = 65); StWSC75, stem WSC at the grain filling stage (Zadoks = 75); TKW, thousand kernel weight; WSC, water-soluble carbohydrate.
