# Supplementary material for: Improving Grain Yield via Promotion of Kernel Weight in High Yielding Winter Wheat Genotypes
Source: Biology (Basel). 2021 Dec 29;11(1):42. doi: 10.3390/biology11010042 (PMC8772892; doi:10.3390/biology11010042)
Supplement: Supplementary file 1 [file biology-11-00042-s001.zip › biology-1492866-supplementary.pdf]

biology

MDPI

Improving Grain Yield via Promotion of Kernel Weight in High Yielding Winter Wheat Genotypes

Supplementary Materials

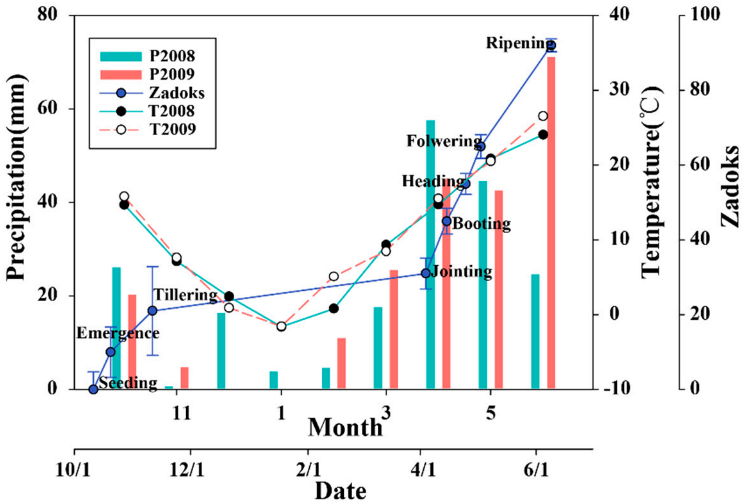

Figure S1. Monthly average temperature and monthly accumulated precipitation for the 2007–2008 and 2008–2009 growing seasons in Tai'an, Shandong, China. Bars in blue and red represent monthly cumulative precipitation during the 2007–2008 and 2008–2009 wheat growing seasons, respectively. Black and white circles represent monthly average temperatures during the 2007–2008 and 2008–2009 wheat growing seasons, respectively. Blue circles represent crop phenology stages on the Zadoks scale [100]. The data were derived from the China Meteorological Data Service Center website (http://data.cma.cn).

Table S1. Year released, pedigree, growing period and cumulative planting area of 15 winter wheat genotypes grown in the Tai'an, Shandong province, China, between 2007 and 2009.

Cultivars	Year released	Pedigree
Taishan 1	1969	Bima 4/Skorospelka L1//Orofen
Jinan 13	1977	Orofen//Huixianhong/Abbondanza
Lumai 15	1985	Tal Yangmai B1/Aimengniu II//104-14
Lumai 14	1986	C149/F_4–530
Lumai 21	1991	Yanzhong 144/Yumai 2
Lumai 23	1991	Lumai 8//Dali'ai
Jinan 17	1993	Linfen 5064/Lumai 13
Jimai 19	1995	Linfen 5064/Lumai 13
Yannong 19	1996	Yan 1933/Shan 82-29
Weimai 8	1998	88-3149/Aus 621108
Jimai20	2000	Lumai 14/Lu 884187
Taishan 23	2002	Yan 881414/Tai 876161
Jimai 22	2003	Ji 935024/Ji 935106
Taishan 223	2004	Ji 5018/Lumai 21
Zhongmai 155	2006	Jimai 19/Lumai 21

Table S2. Agronomic and physiological traits of cultivars grown under field conditions during the 2007–2008 and 2008–2009 wheat growing seasons. The Zadoks growth scale, a numerical code representing various cereal crop growth and development stages, is provided by Zadoks et al. [100].

Classification	Abbreviation	Full name
Agronomic traits	KnSq	Kernels per square meter
	TKW	Thousand-kernel weight
	KnSpk	Kernels per spike
	SpkSq	Spikes per square meter
	KwSpk	Kernel weight per spike
	HI	Harvest index
Physiological traits	Chl65	Chlorophyll content at anthesis stage (Zadoks = 65)
	Pn55	Photosynthesis rate at heading stage (Zadoks = 55)
	Pn65	Photosynthesis rate at anthesis stage (Zadoks = 65)
	Pn75	Photosynthesis rate at grain filling stage (Zadoks = 75)
	CTD65	Canopy temperature depression at anthesis stage (Zadoks = 65)
	CTD75	Canopy temperature depression at grain filling stage (Zadoks = 75)
	LWSC65	Water-soluble carbohydrate in flag leaf at anthesis stage (Zadoks = 65)
	ShWSC65	Water-soluble carbohydrate in flag leaf sheath at anthesis stage (Zadoks = 65)
	StWSC65	Water-soluble carbohydrate in stem at anthesis stage (Zadoks = 65)
	LWSC75	Water-soluble carbohydrate in flag leaf at grain filling stage (Zadoks = 75)
	ShWSC75	Water-soluble carbohydrate in flag leaf sheath at grain filling stage (Zadoks = 75)
	StWSC75	Water-soluble carbohydrate in stem at grain filling stage (Zadoks = 75)
